# Estimating Latent Attentional States Based on Simultaneous Binary and Continuous Behavioral Measures

**DOI:** 10.1155/2015/493769

**Published:** 2015-03-26

**Authors:** Zhe Chen

**Affiliations:** Departments of Psychiatry, Neuroscience and Physiology, School of Medicine, New York University, New York, NY 10016, USA

## Abstract

Cognition is a complex and dynamic process. It is an essential goal to estimate latent attentional states based on behavioral measures in many sequences of behavioral tasks. Here, we propose a probabilistic modeling and inference framework for estimating the attentional state using simultaneous binary and continuous behavioral measures. The proposed model extends the standard hidden Markov model (HMM) by explicitly modeling the state duration distribution, which yields a special example of the hidden semi-Markov model (HSMM). We validate our methods using computer simulations and experimental data. In computer simulations, we systematically investigate the impacts of model mismatch and the latency distribution. For the experimental data collected from a rodent visual detection task, we validate the results with predictive log-likelihood. Our work is useful for many behavioral neuroscience experiments, where the common goal is to infer the discrete (binary or multinomial) state sequences from multiple behavioral measures.

## 1. Introduction

### 1.1. Motivation

In behavioral neuroscience experiments, a common task is to estimate the latent attentional or cognitive state (i.e., the “mind”) of the subject based on behavioral outcomes. The latent cognitive state may account for an internal neural process, such as the motivation and attention. This is important since one can relate the latent attentional or cognitive state to the simultaneous neurophysiological recordings or imaging to seek the “neural correlates” at different brain regions (such as the visual cortex, parietal cortex, and thalamus) [1–4]. Naive determination of such latent states might lead to erroneous interpretations of the result and in some cases even affect the scientific statement. Therefore, it is important to formulate a principled approach to estimate the latent state underlying the behavioral task, such as attention, detection, learning, or decision making [5–9].

In a typical experimental setup of attention task, animals or human subjects are instructed to follow a certain (such as visual or auditory) cue to deliver their attention to execute the task. At each trial, the experimentalist observes the animal's or subject's behavioral outcome (which is of an either binary or multiple choice) as well as the latency (or reaction time) from the cue onset until the execution. However, it shall be cautioned that the observed behavior choice does not necessarily reflect the underlying attentional or cognitive state. For instance, a “correct” behavioral choice can be due to either unattended random exploration or attended execution. In contrast, an “incorrect” behavioral choice can be induced by unattended random exploration or attended yet erroneous decision. Therefore, a simple and direct assignment of behavioral outcomes to attentional states can lead to a false statement or misinterpretation on the behavior. To avoid such errors, it is essential to incorporate* a priori* knowledge or all experimental evidence to estimate the latent state. One direct behavioral measure is the statistics of the latency. Another prior information is the task difficulty and the animal's overall performance. Based on the animal's experiences (naive versus well-trained) or the task difficulty, one can make a reasonable assumption about the dynamics of latent state process. Similar rationale also applies to other cognitive tasks that involves latent state, such as learning, planning, and decision making.

Markovian or semi-Markovian models are powerful tools to characterize temporal dependence of time series data. Markovian models assume history independence beyond the consecutive states (whether it is first-order or high-order dependence), whereas semi-Markovian models allow history dependency; therefore, they are more flexible and they accommodate the Markovian model as special cases. In addition, semi-Markovian models can often be transformed into Markovian models by embedding or augmentation (such as the triplet Markov model) [10]. Typically, Markovian or semi-Markovian models presume stationary probability distributions (for state transition as well as the likelihood function) in time, although this assumption may deviate from the real-life data that often exhibit different degrees of nonstationarity. Despite such deviation, we still believe that Markovian or semi-Markovian models are appropriate for modeling a large class of behavioral data. In addition, statistical models can be adapted to accommodate nonstationarity via online learning, especially for large data sets [11–13].

### 1.2. State of the Art

In the literature, there has been a few works attempting to estimate latent attentional or cognitive states based on simultaneous binary and continuous behavioral measures [15]. In their work, the latent cognitive state was modeled as a continuous-valued random-walk process (which is Markovian). The inference was tackled by an expectation maximization (EM) algorithm [16, 17] based on state space analysis [18, 19].

Alternatively, the attentional state can also be characterized by a discrete or binary variable. Assuming that the attentional state is Markovian or semi-Markovian, one can model the latent process via a hidden Markov model (HMM) [20, 21] or a variable-duration HMM [22] or a hidden semi-Markov model (HSMM) [23–27]. We use the semi-Markovian assumption here. The contribution of this paper is twofold. First, motivated from neuroscience experiments, we formulate the behavioral attention task as a latent state Markovian problem, which may open a way of data analysis in behavioral neuroscience. Specifically, we extend the explicit-duration HMM (or HSMM) to mixed observations (with discrete behavioral outcome and continuous behavioral latency) and derive the associated statistical inference algorithm. This can be viewed as modeling conditionally independent variables with parametric observation distributions in HMM or HSMM [28]. Second, we apply the proposed method to analyze preliminary experimental data collected from a mouse visual attention task.

The rest of the paper is organized as follows. In Section 2, we will present the method that details probabilistic modeling and maximum likelihood inference for the HSMM.  Section 3 presents the results from simulated data as well as experimental data collected from free-behaving mice performing a visual detection task. We conclude the paper with discussions in Section 4.

## 2. Method

### 2.1. Probabilistic Modeling

We formulate the attention process as a hidden semi-Markov chain of two states, where *𝒮* = *S*_1:*T*_ ∈ {0,1} (0: unattended; 1: attended) denotes the latent binary attention variables at trial *t*. Conditioned on the attention state *S*_*t*_, we observe discrete (here, binary) choice outcomes **y** = *y*_1:*T*_ ∈ {0,1} (0: incorrect; 1: correct) and continuous, nonnegative latency measures **z** = *z*_1:*T*_ ∈ *ℝ* ^+^. Unlike the HMM, the HSMM implies that the current state depends not only on the previous state, but also on the duration of previous state [25, 29]. To model such time dependence, we introduce an explicit-duration HMM. Specifically, let *τ*_*t*_ denote the remaining sojourn time of the current state *S*_*t*_. In general, the probability distribution of the sojourn time is

(1) $$\begin{array}{c} P\left(\tau_{t}\;|\;S_{t},\tau_{t-1}\right)=\left\{\begin{array}{cc} I\left(\tau_{t}=\tau_{t-1}-1\right), & \tau_{t-1}>1\\ P\left(\tau_{t}\;|\;S_{t}\right), & \tau_{t-1}=1, \end{array}\right. \end{array}$$

where the indicator function *𝕀*(*τ*_*t*_ = *τ*_*t*−1_ − 1) = 1 if *τ*_*t*_ = *τ*_*t*−1_ − 1 and zero otherwise. In the case of modeling intertrial dependence, the sojourn time *τ*_*t*_ is a discrete random variable *d*; therefore, the explicit duration distribution can be characterized by a matrix **P** = {*p*_*md*_}, where *p*_*md*_ = *p*_*m*_(*d*) (*d* ∈ {1,2,…, *d*_max⁡_}) and the integer *d*_max⁡_ ≤ *T* is the maximum duration possible in any state or the maximum interval between any two consecutive state transitions. Because of the state history dependence, the state transition is only allowed at the end of the sojourn:

(2) $$\begin{array}{c} P\left(S_{t}\;|\;S_{t-1},d\right)=\left\{\begin{array}{cc} I\left(S_{t}=S_{t-1}\right), & 1<d\leq d_{\max}\\ P\left(S_{t}\;|\;S_{t-1}\right), & d=1. \end{array}\right. \end{array}$$

Similar to the standard HMM, the HSMM is also characterized by a transition probability matrix **A** = {*a*_*mn*_} (*m*, *n* ∈ {0,1}), where *a*_*mn*_ = *Pr*⁡(*S*_*t*_ = *m*∣*S*_*t*−1_ = *n*), as well as an emission probability matrix **B** = {*b*_*mk*_}, where *b*_*mk*_ = *P*(*y*_*t*_ = *k*, *z*_*t*_∣*S*_*t*_ = *m*) and *k* ∈ {0,1}. The initial state probability is denoted by ***π*** = *Pr*⁡[*S*_1_]. For all matrices **A**, **B**, and **P**, the sum of the matrix rows is equal to one.

Furthermore, we assume the conditional independence between the binary behavioral measure *y*_*t*_ and the continuous behavioral measure *z*_*t*_; this implies that

(3) bmyt,zt≜P(yt,zt ∣ St=m)=Pr⁡(yt ∣ St=m)P(zt ∣ St=m),

where *P*(*z*_*t*_∣*S*_*t*_ = *m*) is characterized by a probability density function (PDF) parameterized by **ξ**. Since the latency variable is nonnegative, we can model it with a probability distribution with positive support, such as exponential, gamma, lognormal, and inverse Gaussian distribution. For illustration purpose, here we model the latency variable with a lognormal distribution *logn*(*μ*, *σ*):

(4) Pzt=z ∣ St=m=logn(z ∣ μm,σm)≜1z2πσmexp⁡−log⁡z−μm22σm2,

where *z* denotes the univariate latency variable; log⁡(*z*) is normally distributed with the mean *μ*_*m*_ and variance *σ*_*m*_^2^; and **ξ** = {*μ*_*m*_, *σ*_*m*_}_*m*=0_^1^. The lognormal distribution is of the exponential family.

Notes the following.

- Note that it is possible to convert a semi-Markovian chain ({*S*_*t*_}) to a Markovian chain by defining an augmented state {*x*_*t*_} = {*S*_*t*_, *t*_*t*_} and defining a triplet Markovian train (TMC) [10]. The triplet Markov models (TMMs) are general and rich and consist many Markov-type models as special cases.
- If multivariate observations from behavioral measure become available, we can introduce multiple probability distributions (independent case) or multivariate probability distributions (correlated case) to characterize statistical dependency [30].

### 2.2. Likelihood Inference

The goal of statistical inference is to estimate the unknown latent state sequences *𝒮* and the unknown variables {***π***, **A**, **B**, **P**, **ξ**}. Following the derivation of [29], here we present an expectation-maximization (EM) algorithm for simultaneous binary and continuous observations.

We first define a* forward variable* as joint posterior probability of *S*_*t*_ and *τ*_*t*_:

(5) $$\begin{array}{c} \alpha_{t\;|\;t^{\prime }}\left(m,d\right)≜P\left(S_{t}=m,\tau_{t}=d\;|\;y_{1:t^{\prime }},z_{1:t^{\prime }}\right) \end{array}$$

and the marginal posterior probability

(6) $$\begin{array}{c} \gamma_{t\;|\;t^{\prime }}\left(m\right)≜\mathrm{Pr}\left(S_{t}=m\;|\;y_{1:t^{\prime }},z_{1:t^{\prime }}\right)=\overset{d_{\max}}{\underset{d=1}{\sum }}\alpha_{t\;|\;t^{\prime }}\left(m,d\right). \end{array}$$

In addition, we define the ratio of the* filtered* conditional probability over the* predicted* conditional probability:

(7) ρmyt,zt≜αt ∣ t(m,d)αt ∣ t−1(m,d)=P(St=m,τm=d ∣ y1:t,z1:t)P(St=m,τm=d ∣ y1:t−1,z1:t−1)=P(y1:t−1,z1:t−1)P(y1:t,z1:t ∣ St=m,τm=d)P(y1:t,z1:t)P(y1:t−1,z1:t−1 ∣ St=m,τm=d)=bm(yt,zt)P(yt,zt ∣ y1:t−1,z1:t−1)=bm(yt,zt)P(yt ∣ y1:t−1)P(zt ∣ z1:t−1),

where the third step of (7) follows from

(8) Py1:t,z1:t=P(yt,zt,y1:t−1,z1:t−1)=P(y1:t−1,z1:t−1)P(yt,zt ∣ y1:t−1,z1:t−1)

as well as the Markovian property

(9) P(y1:t,z1:t ∣ St=m,τm=d) =P(yt,zt ∣ y1:t−1,z1:t−1,St=m,τm=d)  ·P(y1:t−1,z1:t−1 ∣ St=m,τm=d) =P(yt,zt ∣ St=m)P(y1:t−1,z1:t−1 ∣ St=m,τm=d) ≡bm(yt,zt)P(y1:t−1,z1:t−1 ∣ St=m,τm=d)

and the last step of (7) follows from the conditional independence between *y*_*t*_ and *z*_*t*_.

To compute the predictive probability, we define *r*_1_^−1^ = *P*(*y*_1_, *z*_1_) and

(10) rt−1≜P(yt,zt ∣ y1:t−1,z1:t−1)=∑m,dαt ∣ t−1m,dbmyt,zt=∑mγt ∣ t−1mbmyt,zt,

where *γ*_*t*∣*t*−1_(*m*) = ∑_*d*_ *α*_*t*∣*t*−1_(*m*, *d*). Therefore, the observed data likelihood is given by

(11) $$\begin{array}{c} L=P\left(y_{1:T},z_{1:T}\right)={\left(\overset{T}{\underset{t=1}{\prod }}r_{t}\right)}^{-1}. \end{array}$$

Conditional on the parameters ***θ*** = {***π***, **A**, **B**, **P**, **ξ**}, the expected complete data log-likelihood is written as

(12) E[log⁡P(S1:T,y1:T,z1:T ∣ θ)] =E∑t=1Tlog⁡P(yt ∣ St,θ)+∑t=1Tlog⁡P(zt ∣ St,θ)    +∑m=01log⁡πm+∑t=1Tlog⁡PSt ∣ St−1,τt−1    +∑t=1Tlog⁡P(τt ∣ St,τt−1).

Optimizing the expected complete data log-likelihood with respect to the unknown parameters yields the maximum likelihood estimate.

Similar to [29], we introduce notations for two conditional probabilities:

(13) $$\begin{array}{c} D_{t\;|\;t^{\prime }}\left(m,d\right)≜P\left(S_{t}=m,\tau_{t-1}=1,\tau_{t}=d\;|\;y_{1:t^{\prime }},z_{1:t^{\prime }}\right),\\ T_{t\;|\;t^{\prime }}\left(m,n\right)≜P\left(S_{t-1}=m,S_{t}=n,\tau_{t-1}=1\;|\;y_{1:t^{\prime }},z_{1:t^{\prime }}\right), \end{array}$$

where *𝒟*_*t*∣*t*′_(*m*, *d*) denotes the conditional probability of state *S*_*t*_ starting at state *m* and lasts for *d* time units given the observations; and *𝒯*_*t*∣*t*′_(*m*, *n*) denotes the conditional probability of state transition from *S*_*t*−1_ = *m* to *S*_*t*_ = *n*. Note the consistency holds for ∑_*d*_ *𝒟*_*t*∣*t*′_(*m*, *d*) = ∑_*n*_ *𝒯*_*t*∣*t*′_(*m*, *n*).

To derive the forward-backward updates, we further define a* backward variable β*_*t*_(*m*, *d*) as the ratio of of the smoothed conditional probability *α*_*t*∣*T*_(*m*, *d*) over the predicted conditional probability *α*_*t*∣*t*−1_(*m*, *d*):

(14) βtm,d≜αt ∣ T(m,d)αt ∣ t−1(m,d)=P(St=m,τt=d ∣ y1:T,z1:T)P(St=m,τt=d ∣ y1:t−1,z1:t−1)=P(yt:T,zt:T ∣ St=m,τt=d)P(yt:T,zt:T ∣ y1:t−1,z1:t−1),

where the third equality of (14) follows from

(15) P(St,τt ∣ y1:T,z1:T) =P(St,τt,yt:T,zt:T ∣ y1:t−1,z1:t−1)P(yt:T,zt:T ∣ y1:t−1,z1:t−1) =P(St,τt ∣ y1:t−1,z1:t−1)P(yt:T,zt:T ∣ St,τt)P(yt:T,zt:T ∣ y1:t−1,z1:t−1).

For notation convenience, we define another four sets of random variables:

(16) Etm≜P(St=m,τt=1 ∣ y1:t,z1:t)=αt ∣ t−1m,1ρmyt,zt,Ftm≜P(St+1=m,τt=1 ∣ y1:t,z1:t)=∑nanmEtn,Et∗m≜P(yt:T,zt:T ∣ St=m,τt−1=1)P(yt:T,zt:T ∣ y1:t−1,z1:t−1)=∑dpmdβtm,d,Ft∗m≜P(yt:T,zt:T ∣ St−1=m,τt−1=1)P(yt:T,zt:T ∣ y1:t−1,z1:t−1)=∑namnEt∗n,

where {*ℰ*_*t*_(*m*), *ℱ*_*t*_(*m*)} and {*ℰ*_*t*_^∗^(*m*), *ℱ*_*t*_^∗^(*m*)} represent the forward and backward recursions, respectively. Note that we also have [29]

(17) $$\begin{array}{c} T_{t\;|\;T}\left(m,d\right)=E_{t-1}\left(m\right)a_{mn}E_{t}^{*}\left(n\right),\\ D_{t\;|\;T}\left(m,d\right)=F_{t-1}\left(m\right)p_{m}\left(d\right)\beta_{t}\left(m,d\right). \end{array}$$

### 2.3. EM Algorithm

The EM algorithm for the explicit-duration HMM consists of a forward-backward algorithm (E-step) and the reestimation (M-step). The E- and M-steps are run alternatingly to optimize the expected log-likelihood of the complete data (12).

In the E-step of forward-backward algorithm (note that when *d*_max⁡_ = 1, the forward-backward algorithm reduces to the standard Baum-Welch algorithm used for the HMM.), we can recursively update the forward variable *α*_*t*∣*t*−1_(*m*, *d*) and backward variable *β*_*t*_(*m*, *d*). Specifically, in the forward update,

(18) αt ∣ t−1(m,d) =Ftmpmd+ρmyt−1,zt−1αt−1 ∣ t−2m,d+1,

with an initial value *α*_1∣0_(*m*, *d*) = *π*_*m*_ *p*_*m*_(*d*). And in the backward update,

(19) $$\begin{array}{c} \beta_{t}\left(m,d\right)=\left\{\begin{array}{cc} F_{t+1}^{*}\left(m\right)\rho_{m}\left(y_{t},z_{t}\right), & d=1\\ \beta_{t+1}\left(m,d-1\right)\rho_{m}\left(y_{t},z_{t}\right), & d>1 \end{array}\right. \end{array}$$

with an initial value *β*_*T*_(*m*, *d*) = *ρ*_*m*_(*y*_*T*_, *z*_*T*_) for any *d*. In the end, we obtain the smoothed conditional probabilities *α*_*t*∣*T*_(*m*, *d*) = *α*_*t*∣*t*−1_(*m*, *d*)*β*_*t*_(*m*, *d*), *γ*_*t*∣*T*_(*m*) = ∑_*d*_ *α*_*t*∣*T*_(*m*, *d*), and *𝒟*_*t*∣*T*_(*m*, *d*) and *𝒯*_*t*∣*T*_(*m*, *n*).

In the M-step, we use the smoothed probabilities for reestimating the model parameters $\hat{\theta }$:

(20) $$\begin{array}{c} {\hat{\pi }}_{m}=\frac{\gamma_{1\;|\;T}\left(m\right)}{N_{\pi }},\\ {\hat{a}}_{mn}=\frac{1}{N_{a}}\overset{T}{\underset{t=2}{\sum }}T_{t\;|\;T}\left(m,n\right),\\ {\hat{p}}_{m}\left(d\right)=\frac{1}{N_{p}}\overset{T}{\underset{t=2}{\sum }}D_{t\;|\;T}\left(m,d\right),\\ {\hat{b}}_{mk}=\frac{1}{N_{b}}\overset{T}{\underset{t=1}{\sum }}\gamma_{t\;|\;T}\left(m\right)I\left(y_{t}=k\right)p\left(z_{t}\;|\;{\hat{\mu }}_{m},{\hat{\sigma }}_{m}\right), \end{array}$$

where *N*_*π*_, *N*_*a*_, *N*_*p*_, and *N*_*b*_ are normalizing constants such that the sum of probabilities is equal to one. In addition, the unbiased maximum likelihood estimates of $\left({\hat{\mu }}_{m},{\hat{\sigma }}_{m}^{2}\right)$ in the lognormal distribution are given by

(21) $$\begin{array}{c} {\hat{\mu }}_{m}=\overset{T}{\underset{t=1}{\sum }}w_{t}\left(m\right)\log\left(z_{t}\right),\\ {\hat{\sigma }}_{m}^{2}=\frac{1}{1-\sum_{t=1}^{T}w_{t}^{2}\left(m\right)}\overset{T}{\underset{t=1}{\sum }}w_{t}\left(m\right){\left(\log\left(z_{t}\right)-{\hat{\mu }}_{m}\right)}^{2}, \end{array}$$

where *w*_*t*_(*m*) = *γ*_*t*∣*T*_(*m*)/∑_*n*=0_^1^ *γ*_*t*∣*T*_(*n*).

Upon the algorithmic convergence (the convergence criterion is set as the consecutive log-likelihood increment is less than a small-valued threshold, say 10^−5^), we compute the* maximum a posteriori* (MAP) estimates of the state and duration as

(22) $$\begin{array}{c} \left({\hat{S}}_{t},{\hat{\tau }}_{t}\right)=\arg\underset{\left(m,d\right)}{\max}\;D_{t\;|\;T}\left(m,d\right). \end{array}$$

### 2.4. Model Selection

In practice, the maximum length of state duration *d*_max⁡_ is usually unknown, and we need to estimate the order of the HSMM (since the state dimensionality is fixed here). In statistics, common model selection criteria include the Akaike information criterion (AIC) or Bayesian information criterion (BIC):

(23) $$\begin{array}{c} \text{AIC}=-2\log L+2l,\\ \text{BIC}=-2\log L+l\log T, \end{array}$$

where *ℓ* denotes the total number of free parameters in the model. Alternative order estimator has been suggested [25]:

(24) $$\begin{array}{c} {\hat{d}}_{\max}=\arg\underset{d_{\max}\geq 1}{\min}\left\{-\log L+2c^{2}\log T\right\} \end{array}$$

with *c* = 4*d*_max⁡_^2^.

It shall be emphasized that the AIC and BIC are only asymptotically optimal in the presence of large amount of samples. In practice, experimental behavioral data is often short, and therefore it shall be used with caution or combined with other criteria.

### 2.5. Alternative Parametric Formulation

Previously, we have assumed a nonparametric probability for *p*_*md*_ = *p*_*m*_(*d*) (*d* = 1,…, *d*_max⁡_), which has (*m* − 1)*d*_max⁡_ degrees of freedom. Alternatively, we may assume that the state duration is modeled by a parametric distribution, such as the geometric distribution

(25) $$\begin{array}{c} p_{m}\left(d\right)\equiv \mathrm{Pr}\left(\tau_{m}=d\right)={\left(1-\rho_{m}\right)}^{d-1}\rho_{m}\;\left(d=1,\ldots ,d_{\max}\right), \end{array}$$

where 0 < *ρ*_*m*_ ≤ 1, *𝔼*[*τ*_*m*_] = 1/*ρ*_*m*_, and var[*τ*_*m*_] = (1 − *ρ*_*m*_)/*ρ*_*m*_^2^. In this case, the probabilistic model has *m* degrees of freedom.

For the associated EM algorithm, the E-step remains similar (replacing the calculation of *p*_*m*_(*d*)), whereas the M-step includes additional step to update the parameters of parametric distribution. For instance, in the case of geometric distribution, the parameter *ρ*_*m*_ is updated as

(26) $$\begin{array}{c} {\hat{\rho }}_{m}=\frac{\sum_{t=2}^{T}\gamma_{t\;|\;T}\left(m\right)}{\sum_{t=2}^{T}\sum_{d=1}^{d_{\max}}dD_{t\;|\;T}\left(m,d\right)} \end{array}$$

which is similar to the* methods of moments* in maximum likelihood estimation.

## 3. Results

### 3.1. Simulated Data

*Setup*. In computer simulations, we set the total number of trials as *T* = 100, with the maximum state duration *d*_max⁡_ = 4. We simulate the state sequences and observations using the following matrices:

(27) $$\begin{array}{c} A=\left[\begin{array}{cc} 0.30 & 0.70\\ 0.15 & 0.85 \end{array}\right],\;\;B=\left[\begin{array}{cc} 0.70 & 0.30\\ 0.05 & 0.95 \end{array}\right],\\ P=\left[\begin{array}{cccc} 0.15 & 0.50 & 0.30 & 0.05\\ 0.01 & 0.20 & 0.60 & 0.19 \end{array}\right]. \end{array}$$

**Figure 1:**
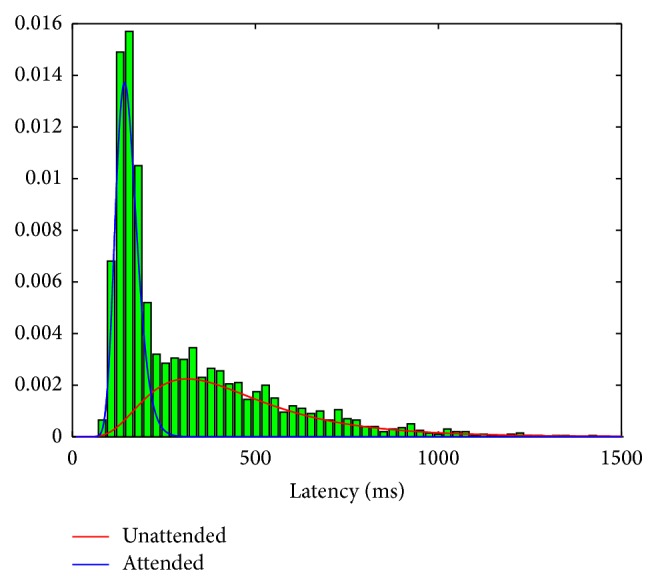
Simulated data: the attended and unattended states have distinct latency distributions (i.e., two modes), as characterized by two lognormal distributions (solid lines).

**Figure 2:**
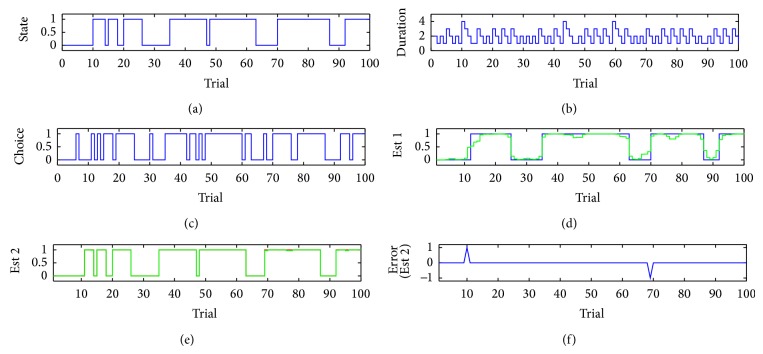
Simulation result. (a) ground truth state sequences. (b) State duration at each trial. (c) Behavioral choice. (d) HSMM estimated state sequence (blue) based on binary behavioral outcomes only (green: state posterior probability). (e) HSMM estimated state sequence (red) based on both binary and continuous behavioral measures (note the MAP and posterior probability nearly overlap). (f) Estimation error between (a) and (e).

The structure of the matrix **P** implies that, for the unattended state, there is a higher probability for state duration of two; for the attended state, the highest probability is for state duration of three. Conditional on the attentional state, the latency variable *z*_*t*_ is assumed to follow a lognormal distribution: *logn*(6,0.5) (for the unattended state) and *logn*(5,0.2) (for the attended state). Two distributions have approximately 13.5% overlap in the area (Figure 1). One realization of simulated latent attentional state sequence *S*_1:*T*_^true^ and behavioral sequence *y*_1:*T*_ are shown in Figure 2. Comparing Figures 2(d) and 2(e) in this illustration, we can see the estimate using both behavioral measures is more accurate and closer to the ground truth (Figure 2(a)). 

*Assessment*. Given the observations *y*_1:*T*_ and *z*_1:*T*_, we run the inference algorithm to estimate the state sequence ${\hat{S}}_{1:T}$. In the simulation where the ground truth is known, the estimation error is defined as

(28) $$\begin{array}{c} \mathrm{err}=\sqrt{\overset{T}{\underset{t=1}{\sum }}{\left|{\hat{S}}_{t}-S_{t}\right|}^{2}}. \end{array}$$

In addition, we define the baseline error as $\text{er}{\text{r}}_{0}=\sqrt{\sum_{t=1}^{T}{\left|y_{t}-S_{t}\right|}^{2}}$ and further compute the relative improvement percentage (RIP):

(29) $$\begin{array}{c} \text{RIP}=\frac{\text{er}{\text{r}}_{0}-\mathrm{err}}{\text{er}{\text{r}}_{0}}\times 100\%. \end{array}$$

A higher value of RIP implies better improvement in the state estimate. For comparison, we run the HSMM-EM algorithm to compute two error rates, one using binary observations *y*_1:*T*_ only (method 1), the other using both binary and continuous observations {*y*_1:*T*_, *z*_1:*T*_} (method 2). We also apply the standard HMM-EM algorithm to analyze the same data using both binary and continuous observation. Furthermore, we consider two scenarios for HSMM. In the first scenario, we assume that the true model order *d*_max⁡_ = 4 is known. In the second scenario, we vary the model order by ±2 from the true model order *d*_max⁡_ (i.e., model mismatch).

We compare the RIP statistic based on 100 independent Monte Carlo runs (although the setup is same, the simulated state sequences and behavioral outcomes are different in each run). The results are summarized in Tables 1 and 2. In both cases, we found that the HSMM (method 2) using both binary and continuous measures yields the best RIP statistic. As expected, when there is a model mismatch from the data, the accuracy of the state estimate degrades.

The results of the HSMM estimate certainly depend on the exact simulation setup. It is expected that when the two-state latency distributions are heavily overlapped (see Figure 1), the estimation error may increase; on the other hand, if the semi-Markovian dynamics can be well approximated by a Markovian dynamics, the difference between the HSMM and HMM will become small. To investigate this issue, we systematically change one of the lognormal distribution (i.e., *μ*_1_) while keeping other parameters unchanged. Essentially, when *μ*_1_ and *μ*_2_ are close in value, there will be a strong overlap in the latency distributions. As seen in Table 3, as *μ*_1_ decreases, the distribution overlap gradually increases; consequently, the performance also gradually degrades. However, the HSMM (method 2) using both binary and continuous behavioral measures still significantly outperforms the HSMM (method 1, comparing Table 1), even in the extreme situation where *μ*_1_ = *μ*_2_ = 5.0.

*Testing the Robustness to Semi-Markovian Assumption*. In addition, we test the robustness of our HSMM and the semi-Markovian assumption for Markovian-driven data. To do that, we generate data from a simple Markovian chain (with a similar setup as before) and then run HMM-EM and HSMM-EM algorithms to compare their RIP. The Monte Carlo results are summarized in Table 4. As seen in this case, the HMM result is slightly more accurate (yet not statistically significant) than the HSMM results because of the nature of Markovian chain; meanwhile, it also confirms the robustness of the HSMM to the Markovian or semi-Markovian assumption.

*Testing the Robustness to Nonstationarity*. Next, we test the the robustness of HSMM and the EM algorithm to nonstationarity. We test two types of nonstationarity: state transition and slow drift of parameter in the likelihood model. In the first case, we consider the state transition in the second half of data sequences are governed by a slightly different probability:

(30) $$\begin{array}{c} A=\left[\begin{array}{cc} 0.50 & 0.50\\ 0.35 & 0.65 \end{array}\right],\;\;P=\left[\begin{array}{cccc} 0.20 & 0.60 & 0.15 & 0.05\\ 0.05 & 0.30 & 0.35 & 0.30 \end{array}\right]; \end{array}$$

yet the other model parameters and *T* remain unchanged. We reestimate the state sequences from simulated data (using HSMM method 2) from 100 independent Monte Carlo runs and obtain the RIP (*d*_max⁡_ = 4) statistic as 0.635 ± 0.022.

In the second case, we allow the parameters of lognormal distribution slightly drift in the second half of data sequences: *μ*_1_ = 5.5, *σ*_1_ = 0.35 (state 1) and *μ*_2_ = 4.5, *σ*_2_ = 0.15 (state 2), yet the other model parameters and *T* remain unchanged. Namely, in the second half, the mean and standard deviation statistics of the latency are reduced for both states and their mode gap is also narrowed. For the new data, we reestimate the state sequences from 100 independent Monte Carlo runs and obtain the RIP (*d*_max⁡_ = 4) statistic as 0.480 ± 0.029.

The result of the first case is not significantly different from that of the stationary setup, and the estimation accuracy in the second case is slightly reduced. The reduction is mostly because the latency variable is more informative in determining the attentional state. Overall, it is concluded that the HSMM method with mixed observations is rather robust to data nonstationarity.

### 3.2. Experimental Data

*Protocol and Animal Behavior*. All experiments were performed in VGAT-cre mice and conducted according to the guidelines of Institutional Animal Care and Use Committee at Massachusetts Institute of Technology and the US National Institutes of Health. All behavioral and physiological data were collected by Dr. Michael Halassa and his team. For details, see [14, 31].

Mice were trained on a visual detection task that requires attentional engagement. Experiments were conducted in a standard modular test chamber. The front wall contained two white light emitting diodes, 6.5 cm apart, mounted below two nose-pokes. A third nose-poke with response detector was centrally located on the grid floor, 6 cm away from the base wall and two small Plexiglas walls (3 × 5 cm), opening at an angle of 20, served as a guide to the poke. All nose-pokes contained an infrared LED/infrared phototransistor pair for response detection. At the level of the floor-mounted poke, two headphone speakers were introduced into each sidewall of the box, allowing for sound delivery. Trial logic was controlled by custom software running on a microcontroller. Liquid reward consisting of 10 *μ*L of evaporated milk was delivered directly to the lateral nose-pokes via a single-syringe pump.

**Figure 3:**
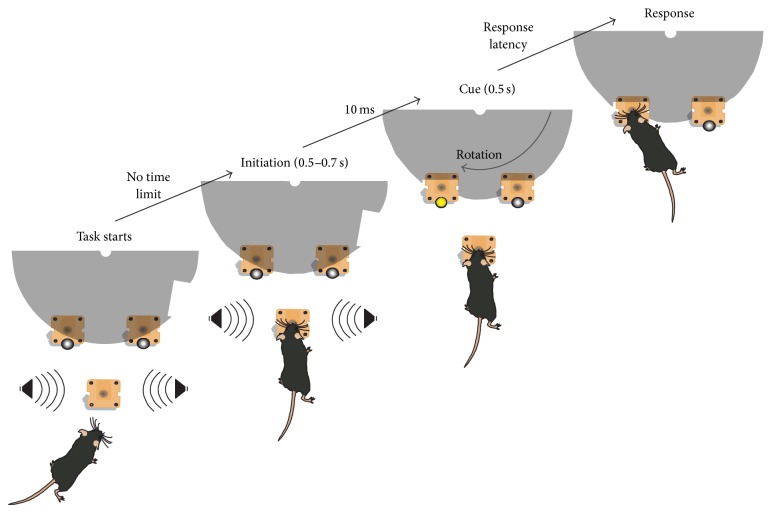
Schematic of the mouse visual detection task (from [14]).

A white noise auditory stimulus signaled the opportunity to initiate a trial. Mice were required to hold their snouts for 0.5–0.7 s into the floor mounted nose-poke unit for successful initiation (stimulus anticipation period). Following initiation, a stimulus light (0.5 s) was presented either to the left or to the right. Responding at the corresponding nose-poke resulted in a liquid reward (10 *μ*L evaporated milk) dispensed directly at the nose-poke (see Figure 3).

*Model Selection and Assessment of Behavioral Data*. The animal behavior (performance and latency) varied at different experimental sessions. The number of trials per session varied between 73 and 152 (mean ± SD: 108 ± 22). The average error rate of the visual detection task across total 20 sessions from two animals is 24 ± 13% (mean ± SD; minimum 6%, maximum 51%). Although the number of states is fixed to two, the model order parameter *d*_max⁡_ remains to be determined. For the two experimental sessions studied here, their basic statistics are shown in Table 5. Notably, for Dataset 1, the average latency is longer (yet statistically nonsignificant, *P* > 0.05, rank-sum test) in incorrect trials than correct trials, whereas for Dataset 2, the average latency is shorter (yet statistically nonsignificant, *P* > 0.05, rank-sum test) in incorrect trials than correct trials.

**Figure 4:**
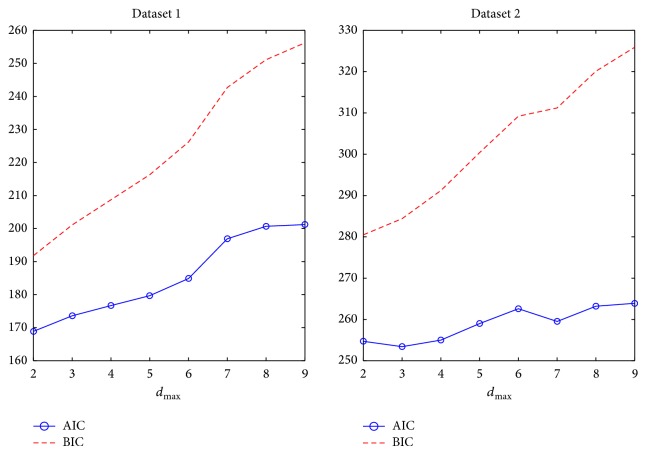
Model selection for *d*_max⁡_ using the AIC and BIC. In Dataset 1, there is no local minimum in both criteria; in Dataset 2, there is a local minimum *d*_max⁡_ = 3 based on the AIC.

**Figure 5:**
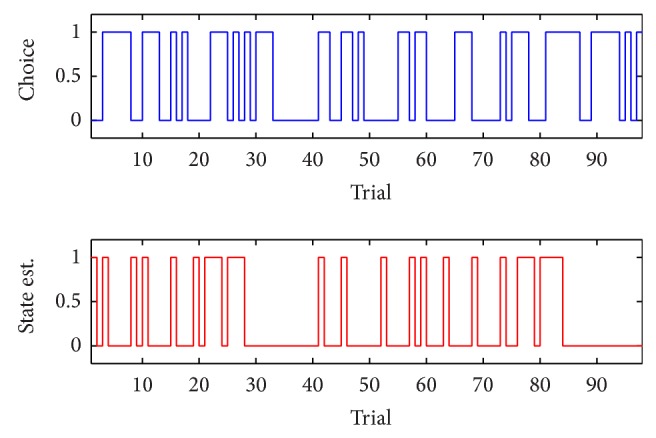
Observed binary behavioral outcomes and estimated attentional state sequences (Dataset 2, using *d*_max⁡_ = 3 based on the AIC). In this example, #{*Y*_*t*_ = 1, *S*_*t*_ = 0} = 33 and #{*Y*_*t*_ = 0, *S*_*t*_ = 1} = 12.

We use 80% data samples for parameter estimation and the remaining 20% for evaluation. In model selection, we compute the AIC and BIC to select a suboptimal *d*_max⁡_. The model selection results for two experimental datasets are shown in Figure 4. Specifically, we found that, for Dataset 1, there is no local minimum within the range of 2 to 9 based on both criteria; whereas for Dataset 2, there is a local minimum *d*_max⁡_ = 3 based on the AIC. As a demonstration, Figure 5 presents the estimated state sequences from Dataset 2 based on *d*_max⁡_ = 3 (Dataset 2). Notably, the estimate of state sequences is nearly identical using *d*_max⁡_ = 5 (if based on the predictive log-likelihood of Table 4). In this case, we observe a relatively big discrepancy between the observed behavioral outcomes and the estimated state sequences. This may be partially due to the high error rate (around 51%) in behavior during this session; notably, unlike most of other sessions, this dataset has an abnormal statistic in that the average error-trial latency is shorter than the average correct-trial latency. Other possible reasons can be the insufficiency of the HSMM or model mismatch or the local maximum of EM optimization. Some of these points will be discussed in the next section.

Since there is no “ground truth” for the attentional state sequences, we also compute the predictive log-likelihood of the 20% held-out data (Table 6). In Table 6, the lowest predictive log-likelihood value is obtained for *d*_max⁡_ = 7 for Dataset 1 and *d*_max⁡_ = 5 for Dataset 2.

## 4. Discussion

In this paper, we have proposed a probabilistic modeling and inference framework for estimating latent attentional states based on simultaneous binary and continuous behavioral measures. The proposed model extends the standard HMM by explicitly modeling the state duration distribution, which yields a special example of the HSMM. The semi-Markovian assumption provides greater flexibility to characterize latent state dynamics.

Estimation of latent attentional states allows us to better interpret the neurophysiological data. Our framework for estimating attentional states is by no means limited by the behavioral measures considered here. In human attention tasks, we may also incorporate other sorts of behavioral measures, such as psychophysics [32].

*Bayesian Inference and Model Extension*. For the simultaneous binary and continuous behavioral measures, we have extended the maximum-likelihood based EM algorithm of [29] for estimating the HSMM parameters, and we have used the AIC or BIC for model selection. The likelihood inference may not yield consistent estimate given a small sample size (in our setup, the sample size *T* is around 100, whereas the degree of freedom in the parameters is around 10–14). This imposes a strong limitation of the likelihood method on model selection in the presence of short behavioral data sequences. An alternative approach is to consider Bayesian inference, either variational or sampling-based Bayesian methods [33–35]. The Bayesian methods may potentially help alleviate the local optimum problem experienced in the likelihood-based EM optimization. Another possibility is to employ the Monte Carlo EM algorithm [26], in which the E-step replaces the traditional Baum-Welch algorithm with reversible jump Markov chain Monte Carlo (MCMC) sampling (where the number of transitions is unknown), and the state estimate is given by the average of Monte Carlo samples [26, 36]. In this case, the estimate obtained from the standard EM algorithm can serve as the initial point for the reversible jump MCMC algorithm [26]. Development of efficient Bayesian inference algorithms will be subject of future work.

The HSMM, or the explicit-duration HMM, is closely related to other work in the literature, such as the sticky HMM [37], sticky HDP-HMM [38], and HDP-HSMM [39]. In these lines of work, the number of states is characterized by a hierarchical Dirichlet process (HDP). Although this is not the issue in our paper (i.e., the number of states is fixed to be two), it may be considered in other multiple-state estimation scenarios. Another possible model extension is to consider a nonparametric Bayesian formulation that allows infinite state duration in HSMM (provided that a large amount of data become available).

*Verification of Experimental Data Analysis*. In experimental data analyses, it is likely that our proposed probabilistic model is insufficient to capture the underlying state dynamics (e.g., nonstationary or switching state dynamics [40]), or that there might be a model mismatch between the empirical latency distribution and the assumed parametric distribution (e.g., lognormal, gamma, or inverse Gaussian). In all analyses, we have witnessed two types of estimation results: one is that the outcome is correct, yet the state is determined to be unattended (i.e., *Y*_*t*_ = 1, ${\hat{S}}_{t}=0$); another is that the outcome is incorrect, yet the state is identified to be attended (i.e., *Y*_*t*_ = 0, ${\hat{S}}_{t}=1$). Since there is no ground truth, it would be reassuring to have another independent measure to corroborate the attentional state estimate. Alternatively, according to the prior knowledge of practical requirement, one may need to formulate a “behaviorally constrained” model and derive a specific “constrained” inference algorithm. This line of research remains to be investigated in the future work.

The ultimate goal of behavioral analysis is to corroborate the neurophysiological data. Therefore, it is also important to verify the results by examining the neural correlates of the attention tasks. This can be in the form of either neuronal firing rate, spike timing or phase synchrony or oscillatory dynamics (power or phase), or LFP evoked potentials, by which one can establish a robust relationship between the attended state and the physiology. In the absence of ground truth, we can rely on the “consistency truth” (condition 1: *Y*_*t*_ = 1, ${\hat{S}}_{t}=1$ and condition 2: *Y*_*t*_ = 0, ${\hat{S}}_{t}=0$) and compare their differences in neural correlates. However, detailed experimental investigation of attentional neural correlates is beyond the scope of current paper.

## Figures and Tables

**Table 1:** Results on the state estimate from the simulated hidden semi-Markovian chain (mean ± sem, computed from 100 independent Monte Carlo runs). The best result is marked in bold font. In contrast, the RIP obtained from the HMM is 0.623 ± 0.025.

	HSMM (method 1)	HSMM (method 2)
RIP (*d*_max⁡_ = 4)	0.084 ± 0.022	0.636 ± 0.025
RIP (*d*_max⁡_ = 2)	0.091 ± 0.019	0.608 ± 0.024
RIP (*d*_max⁡_ = 6)	0.027 ± 0.022	0.611 ± 0.024

**Table 2:** Results on the parameter estimate from the simulated hidden semi-Markovian chain (mean ± sem, computed from 100 independent Monte Carlo runs).

Parameter	HSMM estimate
*μ*_1_ = 6	5.987 ± 0.010
*σ*_1_ = 0.5	0.479 ± 0.007
*μ*_2_ = 5	4.997 ± 0.002
*σ*_2_ = 0.2	0.197 ± 0.002

**Table 3:** Results on the state estimate from the simulated hidden semi-Markovian chain (mean ± sem, computed from 100 independent Monte Carlo runs). The other model parameters remain unchanged. All analyses are based on *d*_max⁡_ = 4.

Mean parameter	Distribution overlap	RIP (HSMM, method 2)
*μ*_1_ = 6.0	13.5%	0.636 ± 0.025
*μ*_1_ = 5.8	22.2%	0.540 ± 0.024
*μ*_1_ = 5.5	39.4%	0.388 ± 0.026
*μ*_1_ = 5.2	54.9%	0.224 ± 0.019
*μ*_1_ = 5.0	58.5%	0.214 ± 0.023

**Table 4:** Results on the state estimate from the simulated hidden Markovian chain (mean ± sem, computed from 100 independent Monte Carlo runs).

	HMM	HSMM (*d*_max⁡_ = 2)	HSMM (*d*_max⁡_ = 3)	HSMM (*d*_max⁡_ = 4)
RIP	0.365 ± 0.021	0.354 ± 0.022	0.351 ± 0.022	0.324 ± 0.022

**Table 5:** Experimental data statistics from two recording sessions.

	Number of trials (correct/error)	Latency (correct)	Latency (error)
Dataset 1	73 (46/27)	6.54 ± 0.44 (s)	7.54 ± 1.31 (s)
Dataset 2	98 (48/50)	7.03 ± 0.85 (s)	6.23 ± 0.71 (s)

**Table 6:** Predicted log-likelihood on the held-out experimental data (using both binary and continuous behavior measures). The greatest value is in bold font.

HSMM	Dataset 1	Dataset 2
*d*_max⁡_ = 2	−22.34	−29.02
*d*_max⁡_ = 3	−28.25	−75.25
*d*_max⁡_ = 4	−31.08	−71.88
*d*_max⁡_ = 5	−30.76	−25.09
*d*_max⁡_ = 6	−30.76	−28.37
*d*_max⁡_ = 7	−21.13	−27.88
*d*_max⁡_ = 8	−25.80	−27.38
*d*_max⁡_ = 9	−25.33	−25.19
